# A socio-legal imperative of domestic violence prohibition in Africa vis-a-vis Nigerian legal structure for sexually abused women

**DOI:** 10.12688/f1000research.132413.1

**Published:** 2023-04-13

**Authors:** Adetutu Deborah AINA-PELEMO, Olusola Joshua Olujobi, EBENEZER TUNDE YEBISI

**Affiliations:** 1 Department of Jurisprudence and International Law, Redeemer’s University, Ede, Osun-State, Nigeria; 2 Department of Public and International Law, Afe Babalola University, Ado Ekiti, Ekiti State, Nigeria; 3 Department of Private and Business Law, College of Law, Afe Babalola University, Ado Ekiti, Ekiti State, Nigeria

**Keywords:** Africa; Custom; Domestic Violence; Gender-Based Violence; Nigerian Legislation; Sexual Abuse Experiences.

## Abstract

Domestic violence is a major issue globally. It is one of the most heinous crimes which has and still results in numerous deaths, still receives the least amount of attention, and its negative influence is being underrated. In Africa, it is customarily acceptable for a woman to be beaten by her husband as a form of discipline, and Nigeria is not an exception. To think otherwise, that it cannot be socially acceptable and legally upheld for a man to beat his wife as a form of discipline, is to deny an existing reality. Section 282 of the Nigerian Penal Code encourages men to beat their wives when necessary. This form of permissible violence is often viewed as a family issue. Hence women are hesitant or reluctant to speak up about their experiences. The stigma that usually follows speaking up or voicing out is better imagined than experienced. This study, therefore, provides credible information on domestic violence incidents in Nigeria and Africa. The methodology utilised is the doctrinal legal research method with reports from existing literature and tertiary data sources such as newspapers and website sources. It discusses legislation enacted to prevent and prohibit domestic violence in Nigeria and how influential they have been on the nation at large. By way of comparative analysis, we examine domestic violence occurrences in some selected African countries and the European continents in relation to Nigeria. It also delves into the violation of the principles of gender equality by some Nigerian customs and traditional practices. This study then makes recommendations on how to address the issue. Through its insightful engagement, this study found, among others, that domestic violence is widespread in Africa and that a national law prohibiting the act and holding perpetrators accountable is not only imperative in Nigeria but across the African continent.

## Introduction

Violence is a concept that has existed since the dawn of civilisation, and it has been a part of the human experience. Violence has numerous conflicting definitions. The World Health Organisation (WHO)

“Defines violence as the intentional use of physical force or power, threatened or actual, against oneself, another person, or a group or community that results in or has a high probability of resulting in injury, death, or psychological harm, mal-development, or deprivation”. ^1^

The United Nations defines “violence against women as any act of gender-based violence that causes or is likely to cause physical, sexual, or mental harm or suffering to women, including threats of such acts, coercion, or arbitrary deprivation of liberty, whether occurring in public or private life”. ^2^ For an act to be regarded as violence, the following must be present: it must be intentional, unwanted, nonessential, and harmful. ^1^ In other words, without these four elements, the act is not necessarily ‘violence’. Violence against women comes in different forms. It could be sexual, physical, and emotional, gender-based, gender discrimination, barbaric traditional practises like forced/early marriage, female genital mutilation (FGM), and many more. It may also include sexual violence and intimate partner violence (IPV) which is also known as domestic violence. Violence against women is a major health and human rights issue. ^3^ It does not only affect their health, it affects their ability to participate fully in society, hinders their enjoyment of sexual and reproductive health and rights, and causes physical and psychological suffering for both the women and their families. ^4^

According to WHO, sexual violence “is any sexual act which includes rape, attempt to obtain sexual act or any other act directed against a person’s sexuality through coercion by any person regardless of their relationship with the victim, in any setting”. ^4^ Sexual violence can be experienced by anyone. However, the majority of those who experience sexual violence are women and children, ^5^ with perpetrators of the act most likely to be either family members, close friends, acquaintances, or strangers. ^6^ Just like violence, sexual violence can take many forms, including but not limited to compelled sex in marriages and dating relationships, stranger rape, sexual harassment, and child abuse. The general aim of this study, using the doctrinal research methodology, is to examine domestic violence as a form of sexual abuse and experiences suffered by women in Africa. It also aims to a spotlight on sexual violence as experienced by women in Nigeria while highlighting relevant Nigerian legislation that prevent or prohibit such sexual experience. This study equally attempts a comparative assessment of legislations and cases laws on domestic violence in some selected countries.”

### Domestic violence as a form of gender-based violence

Violence against women encompasses a wide range of behaviour and acts perpetrated against women, but the most common form being that which occurs between men and their female partners. This form of violence occurs in all cultures and affects women of all ages, irrespective of socio-economic and educational backgrounds. ^7^ Violence definition can be influenced by what obtains in a particular custom and tradition, since there are different traditions and customs. Hence, there will be varieties of the definition of domestic violence. Hence, there is no generally accepted definition of domestic violence. Domestic violence is a type of violence that takes place in an intimate or family relationship. ^8^ Thus it can also be referred to as intimate partner violence (IPV). The perpetrator is a member of the victim's ‘domestic environment’ such as an intimate partner, husband, former intimate partner, family member, friend, or acquaintance. The IPV is defined by how close the relationship is between the perpetrator and the abused victim. Hence, domestic violence will be any destructive behaviour in an intimate relationship where one person tries to dominate and control the other, be it a dating or marital relationship or cohabitation, which causes physical, psychological, or sexual harm to those in that relationship. It includes acts of physical aggression and assault such as hitting or beating with hands or objects, psychological abuse like intimidation, constant belittling or humiliation, forced sexual intercourse, or any other forced or forceful controlling or manipulatively disturbing behaviour which can result into isolating a person from family and friends, tracking their movements, and limiting their access to information or assistance and much more.

Domestic violence is based on inequality between “sexes and anyone”. ^9^ Anyone can be a victim of domestic violence, in other words, men can also be victims of domestic violence, but studies have shown that women and girls are most vulnerable to domestic violence. ^10^ According to data gathered from international sources, men account for 2% of victims of domestic violence committed by a spouse or partner, and women account for 75% and reciprocal violence accounts for 23%. Violence against men and women differ in the sense that men more often experience violence in open spaces, whereas women more often experience violence within close social relationships. ^11^ The terms ‘domestic violence’ and ‘violence’ in the immediate social environment are interchangeably used and refer to adult-on-adult violence. Hence violence by parents or parent proxies against children is not included in the definition of domestic violence rather, it is referred to as child abuse.

Victims of domestic violence go through unimaginable trauma. They must navigate the complexities of an intimate relationship with the offender, as well as the intricacies of a specific trauma and the fear of future assaults by a known assailant. Domestic violence is influenced by unhealthy societal cultural practises or norms which many perpetrators believe they have the right to implement, and in the exercise of these norms along with control tactics on their partners, they really tend to find social support for their acts.

Domestic violence has been visible throughout history. In early Roman society, a woman was considered the husband's property and hence subject to his control. ^12^ A man could beat, divorce, or murder his wife for offences that tarnished his honour or jeopardised his property rights, according to early Roman law. These were regarded as private matters and not subjected to public scrutiny.

In the 15 ^th^ century, ‘the rules of marriage’ of a catholic church charged a husband to stand as a judge of his wife and he was to beat her with a stick upon the commission of an offence. ^13^ According to the ‘rules,’ the beating showed concern for his wife’s soul. Under the common law, a man had a right to beat his wife in order to maintain family discipline, and this was carried out under the principle of ‘rule of thumb’, which allowed a man to beat his wife as long as the stick was no bigger than his thumb. ^14^ Historically in Nigeria, the forefathers also practised patriarchy long before the advent of the colonial masters, but the patriarchal system was not challenged but rather adopted as part of colonial rule. Hence, even after freedom from colonial authority, male supremacy was still being passed down to generations. ^15^ This has made it culturally acceptable for women to regard violence as a form of discipline from their husbands, and they teach their daughters and granddaughters to see it as the same and also as a form of being loved and submissive. These traditions are fairly common in numerous cultures and even in the country's laws.

### An analysis of domestic violence incidents in Africa and other selected countries

Domestic violence is as old as recorded history and has been reported virtually in every society and civilisation. ^12^ Studies have shown that one in three (35%) of women worldwide have experienced either physical and or sexual intimate partner violence or non-partner sexual violence in their lifetime, and as many as 38% of murders of women are committed by an intimate partner. The commonest violence committed against women or girls is intimate partner violence (domestic violence). ^16^ The WHO report of 2005 shows that 36 to 71% of women in other sub-Saharan African countries experience domestic violence. ^17^ A study which examined the issues of domestic violence in other cultures found that 48% of women in Zambia experience intimate or spousal partner violence (domestic violence). ^18^ The same research shows the prevalence of domestic violence in other countries like Colombia, Egypt, India, Cambodia, Peru, and Nicaragua. ^18^

Globally, Africa has the highest rate of cases of violence against women. ^19^ Many African women experience intimate partner abuse and sexual assault early in life because they either marry in their teenage years or have immature sexual relationships. For example, in Southern Africa, at least half of the teenager’s population marries men who are more than five years older than them. ^20^ Zambia and Ethiopia have the highest prevalence rates of violence against wives and intimate partners, with 90% for Zambia and 71% for Ethiopia. ^19^ A report shows that the reasons for the rampage of intimate partner violence in Sub-Saharan Africa could be associated with the polygyny nature of the 16 African countries examined. ^21^ There are higher peculiar IPV happenings among women in polygamous marriages in Malawi, Ethiopia, Burundi, Uganda, Mozambique, Angola, Zimbabwe, and Zambia compared with the women in polygyny in Nigeria and Cameroon. ^21^ This finding indicates that the family structure adopted by most of Sub-Saharan Africa makes women vulnerable to diverse intimate partner violence. The study conducted between 2015-2020 by a group of scholars in Rwanda revealed a decrease in IPV against men and an increase in IPV in women but associated the prevalence with a lack of education due to the available policies in the country that prohibit, prevent, and redress domestic violence. ^22^ According to some scholars, comparably and among 27 Sub-Saharan African countries surveyed, teenage girls between the age of 15 to 19 and young women between the ages of 20 to 24 are found with the highest prevalence of partner violence victimisation with the ratio of 16.1% in Central Africa, 10.4% in Southern Africa, 10.1% in Eastern Africa, and 7.7% in West Africa. ^23^ The fact that sexual assault is frequently combined with physical and psychological abuse by a partner emphasises the special burden that women face in violent relationships.

Until recently, domestic violence was seen as a trivial issue in Ghana, which does not merit any investigation because violence in the home was considered a private matter by the state's law enforcing agencies, and as such, it received or accorded it less importance and attention. ^24^ The victims are discouraged from speaking up because their cases are often disregarded by the police with a piece of advice that it should be settled at home. Domestic violence was perceived as a norm in Ghana, and it was also considered a part of their culture. ^24^ It was and still very difficult for women to admit in public that they are being maltreated by their partners because some found it disgraceful to bring their family matters to the public domain.

In a large-scale analysis of homicide records in many African countries, husbands or partners were responsible for 44.8% of all homicides against women, while women partners were responsible for only 4.4% of homicides against men. ^24^ Injury-related mortality is the most traumatic effect of intimate partner violence, as intimate partner violence is responsible for many of the homicides of women in South Africa. ^11^

### Asian continent with a focus on India as the selected country

Domestic abuse affects 70% of women in India physically, sexually, emotionally, and economically. ^25^ According to India’s National Crime Records Bureau, recorded cases of crime against women rose by 41% (13,892) in 2021 when compared with 2020 (9,782) records, and 31% of the crimes registered were domestic violence. ^26^ Sadly, 65% of men in India believe women should accept such kind of abuse in order to hold the family together and that women sometimes deserve to be battered. ^26^ There is no accurate data on the cases of domestic violence in India because most cases go unreported due to fear of the victim or fear of mockery or embarrassment as well as the pressure not to jeopardise the family’s honour.

It is interesting to know that section 498A of the Indian Penal Code 1860 was established to protect women from the cruelty of their husband or their relatives. ^27^ This section emanated from the case of *ONKAR NATH MISHRA V. STATE,* ^28^ where a woman was beaten by her husband and also maltreated by her in-laws. Article, 14, 15, and 21 of The Protection of Women against Domestic Violence Act 2005 established remedies under civil law for women who are victims of domestic violence, and it prevents domestic violence in society. In spite of this laudable provision and case law, most cases of domestic violence in India go unreported due to the fear of the perpetrators or the fear of mockery or embarrassment, including the pressure not to jeopardise the family’s honour. The research implication of this is that there is no available data on such cases of domestic violence. The Women against Domestic Violence Act 2005 applies to women who are or have been in a relationship with the abuser and are related by consanguinity, marriage or a relationship in the nature of marriage or adoption; relationships with family members living together as a joint family are also covered. In the case of *SARASWATHY V BABU,* ^29^ the Supreme Court held that the victim was entitled to compensation and damages for injuries, including mental torture and emotional distress as a result of the acts of domestic violence committed by her husband. Even those women who are sisters, widows, mothers, single women, or those living with such victims are entitled to get legal protection under the proposed act.

### Domestic violence across the European continent: an analytical overview

The lifetime prevalence of violence in intimate partnerships is reported to be between 10% and 36% in various European countries. ^30^ The percentage of violence against women is low in central Europe because it is normalized, and the victims are usually blamed. ^30^ The government in Europe (judicial system), also have a hand in the large estimate of violence against women because they are of the notion that women are always the offending party. ^30^ In Germany, research conducted by the German Federal Ministry for Family Affairs, Senior Citizens, and Women indicated that violence against women predominantly occurred within their homes. ^31^

A survey was conducted by the European Union Agency for Fundamental Rights (FRA) on the various forms of violence against women among the (28) 28 member states of the European Union. This survey revealed some contradictions in these member states except for Latvia, indicating that 32% of the women have experienced sexual or physical violence by an intimate partner from the age of 15 years old. ^32^ Records of psychological abuse across the European Union showed an average of 43%, while Latvia was 60%. ^32^ Another survey which investigated domestic violence in Switzerland showed that 21% of domestic violence in the country was physical and sexual violence by an intimate partner, while 40% were cases of psychological abuse. ^7^ Equally, the Netherlands and Sweden understudies showed that 22% of the women surveyed have experienced gender-related violence in their lifetime, and approximately 10% of adult women were forced to perform undesirable or uncomfortable sexual acts. The intimate partners of half of these women were identified as perpetrators. ^33^

The FRA survey in Bulgaria showed that the physical and sexual violence ratios of partner violence are higher than the European Union’s average, while non-partner violence percentages for the same questions are lower than the European Union’s average. ^34^ The reason for this is that the criminal justice system prosecutes physical or sexual violence committed by non-partners, while the violence which occurs in a partnership or domestic setting is considered as a family or private affair, thus, it is hard to prosecute. However, in 2005, Bulgaria was one of the first European states to pass a law criminalising domestic violence, namely ‘The Protection against Domestic Violence Act’ way before it became a signatory to the Istanbul convention in 2016. ^34^ The FRA data in Poland highlights how the different cultural contexts toward domestic violence influence responses even within the similar geopolitical space of recent European Union membership and communist histories. ^35^ The percentage of physical and sexual violence by both intimate partners and non-partners is lower in European Union than the average in Poland because gender equality is highly politicised. ^35^ In addition, Poland has resources available for victims of domestic violence, like shelter homes.

According to a 1998 Commonwealth Fund survey, about one-third of American women (31%) have been physically or sexually assaulted by a husband or lover at some point in their lives. ^36^ The National Violence Against Women Survey, which was performed from November 1995 to May 1996, stated that about a quarter of all American women had been raped and/or physically attacked by a current or previous spouse, cohabiting partner, or ‘date’ at a certain stage in their lives. ^5^ In most American states, marital rape is also prohibited and criminalised. In *PEOPLE V LIBERTA* **,** 64 N.Y.2d 152, the Court held that a marriage license should not be used as an avenue for a husband to forcibly rape his wife with impunity.

The Johnny Depp and Amber Heard Case ^37^ is one of the most famous cases of domestic violence. This case, however, shows a downside, while studies have shown that women are most vulnerable to domestic violence. The case reveals that men are most vulnerable to negative use of these statistics. Thus, while women generally are victims of domestic violence, innocent men can be made victims of such domestic violence prevalence, as a man who claims domestic violence will not be believed by a woman who claims so. Again, while the Court has ruled in many instances of domestic violence against men, it will not be disfavoured to rule against a woman who is found guilty of domestic violence.

### A critical review of domestic violence in Nigeria

In Nigeria, intimate partner violence is becoming a widespread threat to the well-being of women in society. Nigerian society is structured along gender lines, resulting in a situation in which women have little or no input in their governance by men. ^38^ Men are considered the dominant group and consequently, have access to large material resources, whilst women a secondary and inferior gender. ^39^

Domestic violence in Africa is heavily influenced by culture and religion, particularly in Nigeria, a nation that is also a multi-ethnic society, dominated. The cultural customs of these major ethnic groups, as well as that of other minority groups, do, however, put women at a disadvantage. ^39^ Traditionally, as in many other African countries, the beating of wives and children is widely accepted as a form of discipline in Nigeria. ^15^ The implication of this is that when parents beat their children, they believe they are imparting discipline to them. The women, by culture, suffer the same fate, as they are seen by their husbands as being prone to indiscipline, and the best way to curtail this is through beating.

Domestic violence against women is often seen as belonging to the private realm in Nigerian society and hence is sheltered from public scrutiny. ^40^ Men use violence as a powerful means of degrading women and are generally at an advantage in terms of how women's lives are restricted and controlled through the use of such violence. ^40^ According to feminist views, marital abuse is inextricably linked to the historical creation of a family in a capitalist society, the division of the public and private worlds, and the specialisation of permissible male and female family responsibilities. ^41^ There is no clear but indirect information on domestic violence in Nigeria because cases of such are rarely contained in the judicial precedents. However, they are found as one of the grounds for divorce cases filed by litigants against their abusers. ^42^ Unfortunately, many women in Nigeria are victims of domestic violence, which is less talked about because it has become acceptable, following the widespread conundrum of the woman that ‘I do not want my marriage to collapse, …’. This recently played out in the widely reported case of a popular Nigerian gospel artist, Osinachi Nwachukwu, who suffered prolonged abuse from her husband before her untimely death. ^43^ Revelations from family, friends, and acquaintances who had knowledge of her domestic violence experiences have emerged; she was advised on several occasions to leave her marriage which she refused to heed and turned down any effort to save the situation. The identified factor for her refusal to speak up or seek refuge was her professed love for her husband and the apparent societal stigma that may affect her now-gained popularity as a gospel singer.

Like Osinachi’s case, the more men assert their dominance as a right backed by cultures and tradition, and the more violence is used against women, breaking the woman’s will, leaving no strength for resistance. It can therefore be said that the most important element reinforcing domestic violence at the personal level is the actuality of control in the social realm.

### Domestic violence in Nigeria through case laws

Domestic abuse cases in Nigeria have increased in recent years, particularly the physical aspect of it. According to legal findings, the South Eastern part of Nigeria has a higher rate of intimate partner violence, accounting for 78.8%, while the prevalence of intimate partner violence was reported to be 29% in South West Nigeria, 42% in Northern Nigeria, and 41% in Southern Nigeria. ^44^ The Nigerian societal customs and religious beliefs concerning women are what reinforce men's abusive practises such as genital mutilation, harmful traditional practises to control women, early girl marriage, one-sided divorce rights in Islamic marriage to men alone, nutritional taboos and other uncouth pregnancy-related practises, to unfavourably widowhood practises and inheritance. ^44^

In the North, a man can marry a child as young as nine years old, subjecting the child to sexual practices that can impair her physical and psychological well-being. ^6^ There have been numerous reports of young females being married, getting pregnant, and becoming victims of domestic violence. Islamic and Christian law also favours the rights of men above those of women. ^45^ In some tribes, when a man dies, the wife is usually suspected and often accused of having a hand in his death. ^46^ This is suggested because most times, while she may not be accused of murdering her husband but she is victimised as being an accomplice after the fact of her husband.

The wife is then subjected to some heinous activities to prove her innocence. For instance, in some places, the widowed woman is forced to drink the coloured water used earlier to wash the corpse of her husband. In most cases, her refusal to undergo these ‘trial by ordeal’ activities is seen as a self-admittance of guilt or a show of culpability in the death of her husband. On the other side, the widowed husband, who has lost his wife, receives more communal commiseration without any investigation as to the cause of the death of his wife, even if it is possible she is a victim of domestic violence. The issue of ‘trial by ordeal’ to prove the innocence of the husband is never in view. It can even be seen as taboo to question the husband over the death of the wife. Again, the custom has outplayed itself.

The practice of marital rape is another deeply rooted social norm in African society. Under Nigerian law, a married woman cannot be raped by her husband. ^47^ The presumption of Nigerian law is that if a man is married to a woman, he is protected from rape charges against her. This immunity applies to husbands in both statutory and customary marriages. It is assumed that a wife has agreed to have sexual intercourse with her partner through marriage and that such consent can only be revoked by a separation agreement or divorce.

### Nigerian cases of marital rape of domestic sexual violence

The provision of the common law rule that a husband cannot rape his wife was clearly adopted into Nigerian jurisprudence. In *R V R*, the Court held that a husband could not be guilty of rape committed by himself upon his lawful wife, for by their mutual matrimonial consent and contract, the wife has given herself up in this kind unto her husband, which she cannot retract. ^48^ Despite the fact that a husband is not criminally accountable for rape, he may be guilty of assault or injury if he uses force or violence to exercise his conjugal right of sexual intercourse. ^40^ In *REX V. CLERK*, the Court held that even while a divorce petition was ongoing, the husband could not be charged with rape against his wife as long as the case had not reached the stage of decree Nisi. ^49^ Decree Nisi is an order by a court of law indicating the date on which a marriage will cease unless a good basis for not granting a divorce is given. ^15^

In *ALAUSA V. ODUSOTE*, a man can be convicted of assault or injury to his wife. As previously stated, because a woman is considered the property of her husband in Nigerian culture, the husband has the authority to deal with her as he deems fit. ^50^ This means that a husband cannot be questioned about the means or methods he chooses to have sex with his wife, and the wife cannot refuse to consent to the means or methods because she is his property. If the wife refuses to consent to the act and the husband proceeds to have his way forcefully, her lack of consent will be inconsequential.

### The religious perspective on marital rape in Nigeria

Under the Islamic religion, a woman cannot refuse to have sex with her husband unless she is ill, menstruating, or has just given birth. ^51^ It is a widespread belief that a woman who refuses her husband's sexual advances will be cursed by Allah's angels for the duration of her refusal. ^51^ This indicates that any violent sexual relationship, be it rape, between a wife and a husband is not classified as unlawful. Equally in the Christian faith, the wife's body belongs to her husband; therefore, a wife is forbidden from denying her husband the pleasure of her body. ^52^ In both faith and even among the traditional deity worshippers, there are no religious injunctions that impose penalties on violators, and these have the power to shape people's way of life and thinking, which may have an impact on their secular laws.

The Nigerian statutory framework, like the Penal code, in Section 282 (2), 1959, does not consider rape as a sexual intercourse between a man and his wife if the wife has reached puberty. The provision of subsection (2) of section 282 is in contradiction with subsection (1) of section 282, which provides for the offence of rape. The contradiction is that subsection (2) established a marital exemption for the crime of rape under the Penal Code. The implication of this is that a man can be guilty of raping his wife if she has not reached puberty. However, due to cultural and religious beliefs, the provision of that law has not been enforced and it might never be. The combined effect of section 357 of the criminal code, CAP C38, Laws of Federation of Nigeria, 2004, which defines rape as having carnal knowledge, and section 6 of the criminal code, which defines carnal knowledge, excluding the sexual relations between the husband and wife and equally states that carnal relation between man and wife is legal, thus legalises marital rape. This notwithstanding, there are other laws in Nigeria which make provision for marital rape, such as the Armed Forces Act and the Sharia Penal Code Law. But these provisions bear much semblance with that of the penal code and the criminal code.

### Gender perspective of domestic violence in Nigeria

There is no distinct gender-specific domestic violence legislation in Nigeria. Women in traditional marriages are immune by virtue of the Evidence Act, which provides some forms of legal remedy in statutory marriages. This leaves those who are married in non-Christian settings (traditional or Muslim marriages) vulnerable to domestic violence. Women in statutory marriages may take comfort in the fact that the Evidence Act provides a legal remedy for physical abuse. Section 180 (c) of the Evidence Act, 2011 Cap E14, Laws of the Federation of Nigeria provides that a man could be criminally accountable for inflicting violence on his wife. This provision has been of less influence due to the fact that in spite of the protection from domestic abuse that it provides, women are still afraid to report incidents of domestic violence to the local authorities with the power to intervene and also because the authorities, when they eventually do intervene do rarely decide in their favour.

Some statutory provisions appear to promote components of domestic abuse, thus undermining the legal battle, such as Section 55 of the Nigerian Penal Code (applicable in Northern Nigeria), which permits husbands to beat their wives for the purpose of correction as long as grievous harm is not caused. According to Section 241 of the Nigerian Penal Code, grievous harm includes; emasculation, permanent loss of sight, hearing or speech, facial disfigurement, deprivation of any limb or joint, bone fracture or tooth dislocation, and other life-endangering harm. Thus, where a husband and wife are subject to customary law that permits wife beating as a form of correction for the wife, the husband can lawfully correct the wife by beating her as long as it does not amount to grievous injury under section 241. Similarly, section 55 (1) (d) of the Penal Code is discriminatory and contradicts the provisions of Section 42 (1) of the 1999 Constitution of the Federal Republic of Nigeria that prohibits discrimination on the basis of sex and gender as well as Article 26 of the International Covenant on Civil and Political Rights which prohibits the discrimination of all persons. The illegality of Section 55 (1) (d) of the Penal Code is compounded by the fact that it gives exclusive right to the husband to discipline his spouse, leaving the spouse helpless with no intervening right. That is, right that is premised on the male gender.

In Nigeria, legislative authorities often ignored cases of intimate partner abuse in the community, believing that women are inferior to males and must live under their rule. According to the Nigerian Federation Laws of 1990, the Criminal Code Act in its Section 353 states that “any individual who unlawfully and indecently assaults any male person commits a felony and is punishable by up to three years in prison. Section 360 of the Criminal Code Act, on the other hand, provides that anyone who unlawfully and indecently assaults a lady or girl is guilty of a misdemeanour and is punishable by imprisonment of two years. Nigerian legislation predominantly demonstrates substantial inequities in the treatment of men and women. For instance, an assault on a man is considered a significant offence and is classified as a ‘felony’, which is a major offence of assault on a woman. On the other hand, it is classified as a misdemeanour or minor felony and is punished less severely than violence against men. This shows the obvious disparity between how the law regards women's abuse when compared to men's abuse.

### A review of the legal framework on domestic violence across states in Nigeria

There have been several initiatives in various states in Nigeria to develop a statutory framework for domestic violence protection. However, only four states in the Federation have passed laws prohibiting the heinous act, ^53^ while several bills are pending in an obviously male-dominated National Assembly. Even at that, the available regulations in these States are short of providing efficient remedies in the absence of any since there is no quick housing or accommodation plan and safe housing for victims during the crisis, with no assurance of prompt execution of policy, no financial security, and proper public awareness. In fact, the laws are yet to be properly tested in the states that have adopted them.

The Violence Against Persons Prohibition (VAPP) Act 2015 is Nigeria's first criminal legislation to outlaw and penalize female genital mutilation, forced eviction of a person's spouse and children, verbal, emotional, and psychological assaults, damaging widowhood practises, political violence, and so on. The VAPP Act also includes a protection order to safeguard domestic abuse victims. The Act prohibits a wide range of acts that were previously acceptable in Nigerian society. The VAPP Act provided a protection order and described it as an order issued by a judge that restrains a person, whether a private or a State actor, from future harmful behaviour toward the victim. ^54^ The statute-barred principle does not apply to a protection order but must be done as prescribed by the Act. The application for such an order can also be filed on behalf of a victim. ^55^ Section 44 of the VAPP Act establishes the National Agency for the Prohibition of Trafficking in Persons and Other Related Matters (NAPTIP) as a regulatory entity tasked with enforcing the provisions of the law and working with relevant parties, including faith-based organisations.

Following the VAPP Act closely in its influence and priority of legislation on domestic violence is the 2007 Lagos State Law on Domestic Violence. For two main reasons, the Lagos State Prohibition against Domestic Violence Law of 2007 is currently regarded as one of the most advanced in Nigeria. ^56^ In addition to criminalising domestic abuse in appropriate situations, it provided a proper civil procedure for dealing with domestic violence events. It is more profound and comprehensive since it targets any victim of domestic abuse, whether women, men, or children, and it also caters to both married and unmarried families. Section 1 of the Law provides that no person shall commit any act of violence against another. This section makes it clear that domestic violence may be committed by any member of the household, and it is not confined to only parties who are married. Section 18 (1) provides for what constitutes domestic violence.

Domestic violence is defined in section 18(g) of the Lagos State Domestic Violence Law as physical abuse, sexual abuse, and exploitation, including but not limited to rape, incest, and sexual assault; starvation; emotional, verbal, and psychological abuse; economic abuse and exploitation; denial of basic education; intimidation; harassment; stalking; hazardous attack including acid both with offensive or poisonous substance; property damage; entry into the complaint's residence. This section also enables a victim of abuse to obtain a protective order in the High or Magistrate Court. This can be lodged by the complainant or any person with his consent who has an interest in the complainant's well-being, such as a counsellor, health care provider, Nigerian Law enforcement officer, social worker, organisation, or teacher. Where the complainant is a minor, mentally handicapped, unconscious, and incapable of consenting for fear of refusal or a person who the Court is satisfied is unable to grant the required consent, another person may apply without the complainant's consent under this same section. Section 2 (5) provides that the application for domestic violence must be filed along with an affidavit and submitted to a court registrar, who must submit it to the Court within 72 hours. However, if the complaint may suffer hardship if the application is not resolved swiftly, it must be brought before a court in chambers.

Another state legislation worthy of note is the Ekiti State Gender-based Violence (Prohibition). The Ekiti State Law prohibiting, Gender-based violence is a combination of Lagos State laws and the Federal Capital Territory's Violence against Persons Prohibition Act 2015. ^56^ It elaborates on violence against people of all genders. Gender-based violence was defined under section 1 in this law as “violence that affects a person or group of people disproportionately because of their gender; any act that causes physical, mental, or sexual harm or suffering; threats of such acts, coercion, or other deprivation of liberty; and all acts of violence that inhibit or neutralize the enjoyment of human rights and fundamental freedoms under general international law or human rights convention as discrimination”. Section 2(b) describes gender-based violence as offences ranging from physical abuse to rape, sexual assault, and violence against women.

The Ekiti State Government has demonstrated that it has one of the most advanced gender-based violence programs in the country. ^57^ The 2019 Ekiti State Gender-Based Violence (Prohibition) Law, the Domestic Violence Protection Order (DVPO), and the 2020 Sexual Violence against Children Compulsory Treatment and Action Care Law are just a few instances of state laws enacted to combat gender-based based violence. So far, the government has guaranteed that these rules are followed and perpetrators penalized. Section 3 (1) (2) (3) provides a penalty for persons who wilfully inflict or causes injury on another through the use of a weapon or persons who commit or aids and abets the commission of an act of violence against another. ^57^^,^^58^ Section 4 provides a penalty for persons who coerce another person to engage in acts which can be detrimental to their physical or psychological health. Section 10 prohibits the ejection of a spouse from their matrimonial home.

It is important to note and discuss the Cross River legislation on domestic violence. There are two major restrictions to the Cross River State Domestic Violence and Maltreatment of Widows Prohibition Law. ^56^ Firstly, it criminalises domestic abuse by making it a crime to subject any woman to any sort of unpleasant treatment or domestic violence, punishable by imprisonment or a fine. The law solely applies to domestic abuse against women. Although it is true that the majority of domestic abuse incidents involve women, the problem cannot be limited to women. As a result, the law is deemed discriminatory. Second, the law limits the definition of domestic violence to cover any abusive use of physical force or energy to inflict damage or injury to a woman at home, in the house, or at any other location.

Ebonyi state followed the same path as cross-river in criminalising domestic abuse. The law on domestic violence in Ebonyi state addresses domestic violence perpetrated between persons in domestic relationships, which was defined as a marital or familial tie between the victim and the respondent. Domestic violence, unlike in cross-river legislation, is characterised as physical assault or abuse, including verbal assaults capable of causing emotional and psychological harm. However, male victims were also excluded under this law.

## Conclusion

Domestic violence is a global problem, particularly in Africa and specifically in Nigeria. In spite of the prevalence of Domestic Violence in these other countries discussed, the majority of them have legislation and a strong regulatory framework that prohibits and penalises it. In addition, they provide resources for domestic violence victims. However, in Nigeria, despite the numerous legal enactments on the prevention, prohibition and redressal of domestic violence in society, domestic violence remains a challenge. Women face inequities in almost every sector of their endeavour. This predicament is intensified by the presence of diverse customary practices such as patriarchy, sex preference, and discrimination in the family, community, and workplace.

Discrimination is common and is fostered by the persistence of preconceptions and customs that contradict human rights principles of natural justice, equity and good conscience. Although the Nigerian government has adopted laws to promote and defend women's rights, no action has been taken, particularly in the area of enhancing or executing those laws, with little progress recorded in areas of public enlightenment. ^59^^–^^61^ In fact, domestic violence is still considered a private affair. Thus, law enforcement agencies continue to limit their involvement in such cases. By preaching that submission to one's husband is required, religious institutions unwittingly contribute to the escalation of domestic violence. The concept of being submissive to one's husband implies respect for him, but African patriarchal society has transformed it into male domination and male oppression. Domestic violence crises are exacerbated by Nigeria's patrilineal and male-dominated system. Even when traditional or communal institutions are successful in preventing domestic violence, they frequently advise the woman to be more respectful of her husband while failing to address the underlying cause. Moreover, most women are unaware of their constitutional and legal rights, leaving them unable to prevent further assaults.

### Recommendations

The recommendations are as follows;

• Barbaric beliefs and practices should be prohibited by the law so as to mitigate domestic violence from the grassroots.
• Domestic violence should be included in the list of Nigerian criminal offences because a unified legal framework could mitigate domestic violence in Nigeria as opposed to the States' fragmented structure.
• Governmental and non-governmental organisations should increasingly educate the public about the dangers of domestic violence through the media or otherwise.
• Perpetrators of such acts should be brought to book in every way possible so as to deter potential ones.
• Religious leaders should be encouraged and educated to advocate against marital violence in their various places of worship, particularly within the Muslim religion.
• Domestic violence victims must be encouraged to break the culture of silence.
• For the protection or advancement of women's rights, the government and non-governmental organisations must organise enlightenment campaigns to raise public awareness.
• Victims should be encouraged to seek medical or external assistance, such as therapists or counsellors, if necessary, to save lives and sanity.
• The younger generation should be taught and encouraged not to abhor such behaviour. This is because a child raised in an abusive home is more likely to imitate such behaviour in the future. Parents indulging in such activities should restrain from doing so and be seen as apologetic about their previous activities

## Data Availability

No data are associated with this article.
