# Supplementary material for: A socio-legal imperative of domestic violence prohibition in Africa vis-a-vis Nigerian legal structure for sexually abused women
Source: F1000Res. 2023 Nov 28;12:397. Originally published 2023 Apr 13. [Version 5] doi: 10.12688/f1000research.132413.5 (PMC10308136; doi:10.12688/f1000research.132413.5)
Supplement: Peer review history

# Reviewer report: Reviewer response for version 5

**DOI:** 10.5256/f1000research.155180.r226343

**Published:** 2023-12-15

**Authors:** Olanike Adelakun

**Affiliations:** 1 School of Law, American University of Nigeria, Yola, Adamawa, Nigeria; 2 School of Law, New York University, New York, New York, USA

This version is a better improvement on the previous version. The author should pay attention to the opening paragraph of the heading 'Legal framework on domestic violence in Nigeria'. The sentence and paragraph appear broken and some information appears to be missing. This affects the flow of the next paragraph. If the statement is a subheading, it is important for the publisher to adopt a font that signifies this or to italicize it. 

 The next heading appears unnecessary because the legal framework should cover the laws that promote and prohibit domestic violence. Creating another subheading on the legal framework that prohibits domestic violence appears confusing and repetitive.

Is the work clearly and accurately presented and does it cite the current literature?

Partly

If applicable, is the statistical analysis and its interpretation appropriate?

Not applicable

Are all the source data underlying the results available to ensure full reproducibility?

Partly

Is the study design appropriate and is the work technically sound?

Partly

Are the conclusions drawn adequately supported by the results?

Partly

Are sufficient details of methods and analysis provided to allow replication by others?

Yes

Reviewer Expertise:

Family Law, Gender Studies, Conflict Studies, Sexual and Reproductive Health Rights, Child and Women Protection.

I confirm that I have read this submission and believe that I have an appropriate level of expertise to confirm that it is of an acceptable scientific standard.

# Reviewer report: Reviewer response for version 4

**DOI:** 10.5256/f1000research.152794.r187457

**Published:** 2023-07-26

**Authors:** Olanike Adelakun

**Affiliations:** 1 School of Law, American University of Nigeria, Yola, Adamawa, Nigeria; 2 School of Law, New York University, New York, New York, USA

While there has been good improvement with the work, I believe that the article could be better improved to fill certain gaps in the literature as I pointed out in the review. Please see comments in document here.

Is the work clearly and accurately presented and does it cite the current literature?

Partly

If applicable, is the statistical analysis and its interpretation appropriate?

Not applicable

Are all the source data underlying the results available to ensure full reproducibility?

Partly

Is the study design appropriate and is the work technically sound?

Partly

Are the conclusions drawn adequately supported by the results?

Partly

Are sufficient details of methods and analysis provided to allow replication by others?

Yes

Reviewer Expertise:

NA

I confirm that I have read this submission and believe that I have an appropriate level of expertise to confirm that it is of an acceptable scientific standard, however I have significant reservations, as outlined above.

# Reviewer report: Reviewer response for version 2

**DOI:** 10.5256/f1000research.149013.r179579

**Published:** 2023-07-03

**Authors:** Olanike Adelakun

**Affiliations:** 1 School of Law, American University of Nigeria, Yola, Adamawa, Nigeria; 2 School of Law, New York University, New York, New York, USA

The article examines domestic violence prevalence in Nigeria and examines some other jurisdictions. 

 There is a need for diligent proofreading of the article. The last three sentences of paragraph 3 and the first 3 sentences of paragraph 4 are the same. There are other repetitions in the work that distort the flow of the work.

 Some parts of the article refer to statistical data and other scholarly work but there were no corresponding references. This needs to be addressed. For instance, the data on the percentage of men and women that experience domestic violence was not referenced.

 The claim that daughters and grand-daughters are taught to normalize domestic violence and GBV should be revised to reflect the position of male children. Does preferential treatment also suggest that male children are taught to believe and exert domination over females? Is that also a way of normalizing domestic violence for male children which contributes to the unbroken chain?

 Reference to domestic violence in Ghana suggests that things have changed but there is no indication throughout the paper on what has changed and if any progress has been made in Ghana. This leaves a gap in the mind of the reader on what that aspect tends to communicate. 

 The claim that there is no data on domestic violence in India should be revisited. There is a difference between lack of data and inadequate or inaccurate data. 

 Reliance on data from a 1995-1998 survey to take a position on domestic violence in America is outdated. Does that suggest that nothing has been done since 1998 that will change the statistics? Also, the discussion of domestic violence in America under a sub-heading that suggests that the section is focused on Europe is misleading. 

 On page 7, the position that domestic violence is a ground for divorce in Nigeria should be changed to reflect that domestic violence can be claimed to prove that a marriage has broken down irretrievable. The only ground for divorce of statutory marriage is that the marriage has broken down irretrievably. Also, the reference on this note (42) should be corrected to section 15(2)(e) and not 15(1)(e) of the MCA.

 The claim of the one-sided right to divorce in Islamic marriage should be reviewed. The authors may argue on the imbalance or inequality in divorce rights and not one-sided rights. It is important to compare the concept of talaq and khul to effectively make this argument. 

 The author's position on marital rape needs to be updated. Though, under sections 6 & 357 of the Criminal Code Act and section 382(2) of the Penal Code Act, marital rape is not recognized as a crime. However, the authors should evaluate this argument in line with VAPPA, ACJA, and the Constitution. Section 42(2) of the VAPPA is important in resolving conflict of laws in this regard. Though the authors made reference to this towards the end of the paper, this is a very important argument and should flow appropriately. 

 On the gender perspective of domestic violence in Nigeria, the authors needs to review the general application of the VAPPA to all persons irrespective of the nature of the marriage entered into. 

 The claim that the authorities often ignored cases of IPV in Nigeria is misleading. Several reports from the appropriate government agencies, NGOs, and INGOs show otherwise. Institutional restructuring across Nigeria also shows otherwise. For instance, in recent times, many police stations now have a gender desk with officers constantly trained to empower them in responding to domestic violence and GBV cases. Advocacy work is also evident in this area. The effectiveness and progress made may be worth evaluating. 

 The authors claim that only four states in Nigeria have enacted laws on domestic violence whereas the corresponding reference shows about 8 states. Presently, almost 20 states have taken legislative and policy steps directly and indirectly on domestic violence in Nigeria. There is a need to refer to current literature on this aspect in order to provide an up to date information. 

 The conclusion of a scholarly work should basically give a summary of what has been done and not introduce something new that was not discussed in the paper. The authors should reflect on this and avoid introducing new elements in the conclusion. Also, avoid making reference to other works in the conclusion and recommendations since these parts are expected to be solely your work. 

 Finally, the authors' voices were missing from the paper. There is a need to clearly take a position that reflects the stand of the authors.

Is the work clearly and accurately presented and does it cite the current literature?

Partly

If applicable, is the statistical analysis and its interpretation appropriate?

Not applicable

Are all the source data underlying the results available to ensure full reproducibility?

Partly

Is the study design appropriate and is the work technically sound?

Partly

Are the conclusions drawn adequately supported by the results?

Partly

Are sufficient details of methods and analysis provided to allow replication by others?

Yes

Reviewer Expertise:

Family Law, Gender Studies, Conflict Studies, Sexual and Reproductive Health Rights, Child and Women Protection.

I confirm that I have read this submission and believe that I have an appropriate level of expertise to confirm that it is of an acceptable scientific standard, however I have significant reservations, as outlined above.

# Response

**Published:** 2023-07-07

**Authors:** OLUSOLA JOSHUA OLUJOBI

**Affiliations:** PUBLIC AND INTERNATIONAL LAW, Afe Babalola University, Ado Ekiti, Ekiti, Nigeria

**THE ARTICLE EXAMINES DOMESTIC VIOLENCE PREVALENCE IN NIGERIA AND EXAMINES SOME OTHER JURISDICTIONS**

 There is a need for diligent proofreading of the article. The last three sentences of paragraph 3 and the first 3 sentences of paragraph 4 are the same. There are other repetitions in the work that distort the flow of the work.

**Authors Responses**

**Many thanks for the pain, time and effort used to review our paper, and thanks for the candid comments and suggestions to make the work robust. God bless you and your family. Once again,** **thank you for your efforts** **.** **Diligent proofreading of the article has been meticulously and painstakingly done. The last three sentences of paragraph 3 and the first 3 sentences of paragraph 4 which appeared the same have been corrected.  Also, other repetitions in the work that distort the flow of the work have been overhauled diligently. Thanks, sir. It was done in compliance with the reviewer's comments. Please see pages 1-2.**

 Some parts of the article refer to statistical data and other scholarly work but there were no corresponding references. This needs to be addressed. For instance, the data on the percentage of men and women that experience domestic violence was not referenced.

**Authors Responses**

**Once again, thank you for your efforts. Statistical data and other scholarly work mentioned are now referenced. For instance, the data on the percentage of men and women that experience domestic violence is now referenced. Thanks, sir. The correction was done in compliance with the reviewer’s comments. Please see pages 1-20.**

 The claim that daughters and granddaughters are taught to normalize domestic violence and GBV should be revised to reflect the position of male children. Does preferential treatment also suggest that male children are taught to believe and exert domination over females? Is that also a way of normalizing domestic violence for male children which contributes to the unbroken chain?

**AUTHORS RESPONSE**

**Thank you for your efforts. The claim that daughters and granddaughters are taught to normalize domestic violence has been revised to reflect the position of male children. Preferential treatment which suggests that male children are taught to believe and exert domination over females appear to normalise domestic violence for male children which has contributed to the unbroken chain of violence. This has made it culturally acceptable for women to regard violence as a form of discipline from their husbands, and they teach their daughters and granddaughters to see it as the same and also as a form of being loved and submissive.  While on the sons' and grandsons’ perspective, patriarchy has led to a preference for a male child over a female which boosts their ego.  Furthermore, male children have been thought religiously and customarily to assert authority in every way possible even if it leads to domestic violence over their spouse.** **Thanks, sir. The correction was done in compliance with the reviewer's comments. Please see page 5.**

 Reference to domestic violence in Ghana suggests that things have changed but there is no indication throughout the paper on what has changed and if any progress has been made in Ghana. This leaves a gap in the mind of the reader on what that aspect tends to communicate.

**AUTHORS RESPONSE**

**Many thanks for the pain, time and effort used to review our paper, and thanks for the candid comments and suggestions to make the work robust. God bless you and your family. Once again, thank you for your efforts. The reference to domestic violence in Ghana which suggests things have changed has been included in the paper with the progress made in Ghana.** **A study conducted by Adjah and Agbenafle reported that the prevalence of domestic violence remains unacceptably high in Ghana despite the governmental provision of shelter for domestic abuse victims, the adoption of the Declaration on Elimination of Violence Against Women (DWVAW) convention, and the creation of domestic violence and victims' Support Unit of the Ghana Police service. The correction was done in compliance with the reviewer’s comments. Please see page 6.**

 The claim that there is no data on domestic violence in India should be revisited. There is a difference between lack of data and inadequate or inaccurate data.

**AUTHORS RESPONSE**

**Many thanks for the pain, time and effort used to review our paper, and thanks for the candid comments and suggestions to make the work robust. God bless you and your family. Once again, thank you for your efforts. The claim that there is no data on domestic violence in India has been revisited. The statement there is an inadequate date or inaccurate data has been corrected. The correction was done in compliance with the reviewer’s comments. Please see page 7**

 Reliance on data from a 1995-1998 survey to take a position on domestic violence in America is outdated. Does that suggest that nothing has been done since 1998 that will change the statistics? Also, the discussion of domestic violence in America under a sub-heading that suggests that the section is focused on Europe is misleading.

**AUTHORS RESPONSE**

**Many thanks for the pain, time and effort used to review our paper, and thanks for the candid comments and suggestions to make the work robust. God bless you and your family. Once again, thank you for your efforts.** **According to a 1998 Commonwealth Fund survey, about one-third of American women (31%) have been physically or sexually assaulted by a husband or lover at some point in their lives.** **The National Violence Against Women Survey, which was performed from November 1995 to May 1996, stated that about a quarter of all American women had been raped and/or physically attacked by a current or previous spouse, cohabiting partner, or ‘date’ at a certain stage in their lives.^^** **Firstly, the U.S. Congress entreated a multi-year strategy to eradicate and address issues of violence against women and girls in 2012 and the strategy was updated in 2016. Meanwhile, the violence against Women Reauthorisation Act of 2013 prevents GBV and any forms of violence in marriage. However, the said strategy was a national plan to end violence in the United States, and it was adopted in 2022.** **The correction was done in compliance with the reviewer’s comments.** **Please see page** **8.**

 On page 7, the position that domestic violence is a ground for divorce in Nigeria should be changed to reflect that domestic violence can be claimed to prove that a marriage has broken down irretrievable. The only ground for divorce of statutory marriage is that the marriage has broken down irretrievably. Also, the reference on this note (42) should be corrected to section 15(2)(e) and not 15(1)(e) of the MCA.

**AUTHORS RESPONSES**

**Many thanks for the pain, time and effort used to review our paper, and thanks for the candid comments and suggestions to make the work robust. God bless you and your family. Once again, thank you for your efforts.** **the reference on a note (42) has been corrected to read to section 15(2)(e) of the MCA.**

**However, they are found as one of the grounds for divorce cases filed by litigants against their abusers, and domestic violence is only a ground to show that the marriage has broken down irretrievably. In section (16) (ii) of the Matrimonial Causes Act, a ground stated for a marriage to be deemed to have broken down irretrievably, which could lead to the dissolution of marriage or the decree Nisi of the court to the permanent separation of the couple or parties’ section 15(2)(e) of the MCA. The correction was done in compliance with the reviewer’s comments. Please see page 10.**

 The claim of the one-sided right to divorce in Islamic marriage should be reviewed. The authors may argue on the imbalance or inequality in divorce rights and not one-sided rights. It is important to compare the concept of talaq and khul to effectively make this argument.

**AUTHORS RESPONSES**

**The claim of the one-sided right to divorce in Islamic marriage has been reviewed. The authors have now argued on the imbalance or inequality in divorce rights and compared the concept of talaq and khul.** **The Nigerian societal customs and religious beliefs concerning women are what reinforce men's abusive practises such as genital mutilation, harmful traditional practises to control women, early girl marriage, nutritional taboos and other uncouth pregnancy-related practises, unfavourably widowhood practises and inheritance, one-sided divorce rights under Islamic marriage system,** **considering two main principles on divorce under this religion, the ‘ *Talaq*’ and ‘ *Khul*’, which govern the way a man and a woman can divorce their spouse. These principles favour man than women. *Talaq* permits the man to divorce his wife with payment of the *Mahr* if the marriage has been consummated, but if not, he pays half. The *Khul* allow the wife to untie the nut between her and the husband but at a price.** **The correction was done in compliance with the reviewer’s comments.  Please see page 10.**

 The author's position on marital rape needs to be updated. Though, under sections 6 & 357 of the Criminal Code Act and section 382(2) of the Penal Code Act, marital rape is not recognized as a crime. However, the authors should evaluate this argument in line with VAPPA, ACJA, and the Constitution. Section 42(2) of the VAPPA is important in resolving conflict of laws in this regard. Though the authors referred to this towards the end of the paper, this is a very important argument and should flow appropriately.

**AUTHORS RESPONSES**

**The author's position on marital rape has been updated.** **By virtue of the provisions of Criminal code Act and Penal Code Act of Nigeria Marital rape is not regarded as a punishable offence. However, the Constitution of the Federal Republic of Nigeria (CFRN) provides that everyone deserves a level of respect and freedom from all forms of torture or degrading treatment. Rape could indirectly fall within the ambit of disrespect to the dignity of a person whether he or she is lawfully married to the rapist.  Similarly, the Violence Against Persons’ Act of the Federal Capital Territory, Abuja, 2015 provides that a body shall be appointed to manage issues related with or incidental to domestic violence and shall report to the Federal Government annually and the government shall forward such reports to the government parastatal in charge of Bureau statistics.**

**In addition, the Administration of Criminal Justice Act permits married women to seek redress by way of criminal proceedings against anybody including their husbands to ensure their personal security and property protection.**

**In essence, though some specific laws clearly do not recognize marital or spousal rape as an offence, but the CFRN which is the foundation of all other laws, ACJA, and the VAPPA empower women to defend and seek remedies against their husbands in case of any criminal violation of their private or public rights.**

**This important argument now flows appropriately.** **The correction was done in compliance with the reviewer’s comments.  Please see page 11.**

 On the gender perspective of domestic violence in Nigeria, the authors need to review the general application of the VAPPA to all persons irrespective of the nature of the marriage entered into. 

**AUTHORS RESPONSES**

**The authors now reviewed the general application of the VAPPA to all persons irrespective of the nature of the marriage.** **The Violence Against Persons Prohibition (VAPP) Act 2015 is Nigeria's first criminal legislation to outlaw and penalize female genital mutilation, forced eviction of a person's spouse and children, verbal, emotional, and psychological assaults, damaging widowhood practices, political violence, and so on. It is important to state that this Act applies to everyone irrespective of their biological sex, gender, and nature of marriage. The VAPP Act also includes a protection order to safeguard domestic abuse victims. The Act prohibits a wide range of acts that were previously acceptable in Nigerian society. The VAPP Act provided a protection order and described it as an order issued by a judge that restrains a person, whether a private or a state actor, from future harmful behaviour toward the victim.^^** **The statute-barred principle does not apply to a protection order but must be done as prescribed by the Act. The application for such an order can also be filed on behalf of a victim.** **Section 44 of the VAPP Act establishes the National Agency for the Prohibition of Trafficking in Persons and Other Related Matters (NAPTIP) as a regulatory entity tasked with enforcing the provisions of the law and working with relevant parties, including faith-based organisations.** **The correction was done in compliance with the reviewer’s comments.  Please see page 13.**

 The claim that the authorities often ignored cases of IPV in Nigeria is misleading. Several reports from the appropriate government agencies, NGOs, and INGOs show otherwise. Institutional restructuring across Nigeria also shows otherwise. For instance, in recent times, many police stations now have a gender desk with officers constantly trained to empower them in responding to domestic violence and GBV cases. Advocacy work is also evident in this area. The effectiveness and progress made may be worth evaluating.

**AUTHORS RESPONSES**

**The claim that the authorities often ignored cases of IPV in Nigeria has been corrected to read: In Nigeria, legislative authorities often ignored cases of intimate partner abuse in the community, believing that women are inferior to males and must live under their rule.** **The correction was done in compliance with the reviewer’s comments.  Please see page 12.**

 The authors claim that only four states in Nigeria have enacted laws on domestic violence whereas the corresponding reference shows about 8 states. Presently, almost 20 states have taken legislative and policy steps directly and indirectly on domestic violence in Nigeria. There is a need to refer to current literature on this aspect to provide up-to-date information.

**UTHORS RESPONSES**

**The authors claim that only four states in Nigeria have enacted laws on domestic violence and have been corrected to read 8 states. Presently, almost 20 states have taken legislative and policy steps directly and indirectly on domestic violence in Nigeria. Current literature has been referred to on this aspect to provide up-to-date information.** **There have been several initiatives in various states in Nigeria to develop a statutory framework for domestic violence protection. However, (33) thirty-three states of the Federation, have domesticated the VAPP Act as of 2022.) while several bills are pending in a male-dominated National Assembly.** **The correction was done in compliance with the reviewer’s comments.  Please see pages 13-14.**

 The conclusion of a scholarly work should give a summary of what has been done and not introduce something new that was not discussed in the paper. The authors should reflect on this and avoid introducing new elements in the conclusion. Also, avoid referring to other works in the conclusion and recommendations since these parts are expected to be solely your work.

**AUTHORS RESPONSES**

**The conclusion has been reworked to give a summary of what has been done only and not introduce something new that was not discussed in the paper. The work is now solely the authors’ work.** **The correction was done in compliance with the reviewer’s comments.  Please see pages 14-15.**

 Finally, the authors' voices were missing from the paper. There is a need to take a position that reflects the stand of the authors.

**AUTHORS RESPONSES**

**The authors' voices are now conspicuous in the paper. A clear position that reflects the stand of the authors is crystal clear in the paper. Many thanks for the pain, time and effort used to review our paper, and thanks for the candid comments and suggestions to make the work robust. God bless you and your family. Once again, thank you for your efforts** **The correction was done in compliance with the reviewer’s comments.  Please see pages 1-20.**

# Reviewer report: Reviewer response for version 2

**DOI:** 10.5256/f1000research.149013.r179590

**Published:** 2023-06-28

**Authors:** Paul Ejembi

**Affiliations:** 1 Department of International Law and Jurisprudence, Faculty of Law, Benue State University, Makurdi, Benue, Nigeria

The article presents a panoramic perspective of the socio-legal imperative of domestic violence prohibition in the African Continent with particular reference to Nigeria. It makes a comparative analysis of the occurrences of the menace of domestic violence in different socio-cultural settings particularly in India and Nigeria which have similar socio-political characteristics.

 The article is clear, easy to understand and unequivocally addresses the social and legal issues it aims to achieve. It is my calculated opinion that the article is publishable and definitely contributes to knowledge especially as domestic violence in Nigeria is concerned.

 It is, however, suggested that the recommendations articulated by the authors should be written in paragraphs or enumerated instead of the dotted symbols used.

Is the work clearly and accurately presented and does it cite the current literature?

Yes

If applicable, is the statistical analysis and its interpretation appropriate?

Not applicable

Are all the source data underlying the results available to ensure full reproducibility?

Yes

Is the study design appropriate and is the work technically sound?

Yes

Are the conclusions drawn adequately supported by the results?

Yes

Are sufficient details of methods and analysis provided to allow replication by others?

Yes

Reviewer Expertise:

Public international law, Human rights, Gender and the law, Jurisprudence, International and comparative disability law, legal theory

I confirm that I have read this submission and believe that I have an appropriate level of expertise to confirm that it is of an acceptable scientific standard.

# Response

**Published:** 2023-06-28

**Authors:** OLUSOLA JOSHUA OLUJOBI

**Affiliations:** PUBLIC AND INTERNATIONAL LAW, Afe Babalola University, Ado Ekiti, Ekiti, Nigeria

Many thanks for the pains, efforts, time you took to review our work and for your candid comments and suggestions to make the work robust. God bless you and your family. Once again, thank you  for your efforts.

 The recommendations section has suggested by the reviewer is now written in paragraphs or enumerated instead of the dotted symbols used earlier. Thank you.

# Reviewer report: Reviewer response for version 2

**DOI:** 10.5256/f1000research.149013.r176483

**Published:** 2023-06-13

**Authors:** Chisaa Onyekachi Igbolekwu

**Affiliations:** 1 Centre for Gender, Humanitarian and Development Studies, Redeemer's University, Ede, Osun, Nigeria

The revisions made are in order, kindly index the article. Thank you.

Is the work clearly and accurately presented and does it cite the current literature?

Yes

If applicable, is the statistical analysis and its interpretation appropriate?

Yes

Are all the source data underlying the results available to ensure full reproducibility?

Yes

Is the study design appropriate and is the work technically sound?

Yes

Are the conclusions drawn adequately supported by the results?

Yes

Are sufficient details of methods and analysis provided to allow replication by others?

Partly

Reviewer Expertise:

Medical Sociology, Demography, Gender issues, especially in women's health, fertility and GBV

I confirm that I have read this submission and believe that I have an appropriate level of expertise to confirm that it is of an acceptable scientific standard.

# Reviewer report: Reviewer response for version 1

**DOI:** 10.5256/f1000research.145329.r169561

**Published:** 2023-05-17

**Authors:** Chisaa Onyekachi Igbolekwu

**Affiliations:** 1 Centre for Gender, Humanitarian and Development Studies, Redeemer's University, Ede, Osun, Nigeria

**Abstract**

 Secondary data and not tertiary is preferably used.

 The result or findings section of the abstract should incorporate more findings made by the study.

**Introduction**

 The introduction of the paper was quite elaborate and well-articulated. However there is need for a proper gap identification that will help the readers understand the focal point of study.

**Objective of this study**

 There is need for clarity on the objective of the study as that will help to guide the focus of the study.

**An analysis of domestic violence incidents in Africa and other selected countries**

 Under this subheading, the authors projected legal framework around domestic violence as it were in the traditional societies and failed to project the current move by different countries to ameliorate the menace of domestic violence.

 Domestic violence is no longer a hush-hush issue as it were in the past. The culture of silence over domestic violence has been broken, many people are beginning to speak out. Similarly the paper should also look into the roles of some of the treaties many of the aforementioned African counties are signatory to that has also helped to reduce domestic violence.

 Similarly some states in Nigeria have also adopted more stringent sanctions and panel codes for some types of gender based violence, the functionality and effectiveness of some of the laws should also be interrogated.

**Asian continent with a focus on India as the selected country**

 Under this subheading and in the previous one, the authors should justify the reasons for selection of India among all the Asian countries. Similarly, the criteria used for the selection of some of the countries under discuss should also be made clear.

 The lack of clarity in the justification and criteria could also be because the authors did not dedicate a subheading to address the methodology used in the study, it was only mentioned in the abstract. Therefore there is need to dedicate a section of the manuscript to explain the methods.

**Summary of review**

 The study is well articulated, comprehensive, detailed and addressed a very pressing social problem that cannot be over emphasized in Africa and especially Nigeria.

 The conclusion was precise and drawn from the research findings and the recommendations are apt, Approve for publications after minor corrections,

Is the work clearly and accurately presented and does it cite the current literature?

Yes

If applicable, is the statistical analysis and its interpretation appropriate?

Yes

Are all the source data underlying the results available to ensure full reproducibility?

Yes

Is the study design appropriate and is the work technically sound?

Yes

Are the conclusions drawn adequately supported by the results?

Yes

Are sufficient details of methods and analysis provided to allow replication by others?

Partly

Reviewer Expertise:

Medical Sociology, Demography, Gender issues, especially in women's health, fertility and GBV

I confirm that I have read this submission and believe that I have an appropriate level of expertise to confirm that it is of an acceptable scientific standard, however I have significant reservations, as outlined above.

# Response

**Published:** 2023-05-21

**Authors:** OLUSOLA JOSHUA OLUJOBI

**Affiliations:** PUBLIC AND INTERNATIONAL LAW, Afe Babalola University, Ado Ekiti, Ekiti, Nigeria

We the authors deeply appreciated the reviewer for taking the time to review our article. The

 comments and observations are valued. Our responses to some of the issues raised are

 presented below:

**Abstract**

 Secondary data and not tertiary is preferably used. The result or findings section of the abstract should incorporate more findings made by the study.

**Response: **The authors adopted the doctrinal research methodology, which is majorly used by legal scholars, lawyers and judges researching in law to draw out new legal theories logically. It involves the qualitative research approach where data was drawn from primary and secondary sources via consultation with legal instruments, such as legislation, case laws, statutes, and policies. The use of secondary data is not necessary for the study. The robust methodology is one of the areas that F1000Research emphasized for article publication consideration. Thank you exceedingly for the pain, time and efforts to review our paper, candid comments and suggestions to make the work robust. 

**Introduction**

 The introduction of the paper was quite elaborate and well-articulated. However, there is a need for a proper gap identification that will help the readers understand the focal point of the study.

**Response:  **Proper identification of the ****gaps to help the readers understand the focal point of the study has been discussed and identified succinctly.Thank you exceedingly for the pain, time and efforts to review our paper, candid comments and suggestions to make the work robust.

 The objective of this study: There is a need for clarity on the objective of the study as that will help to guide the focus of the study.

**Response: **The general aim or objective of this study, using the Doctrinal research methodology, is to examine domestic violence as a form of sexual abuse and experiences suffered by women in Africa. It also aims to a spotlight on sexual violence as experienced by women in Nigeria while highlighting relevant Nigerian legislation that prevents or prohibits such sexual experience. This study equally attempts a comparative assessment of legislation and case laws on domestic violence in some selected countries. The aim of the study has been brought out with clarity to guide the focus of the study. Thank you exceedingly for the pain, time and efforts to review our paper, candid comments and suggestions to make the work robust. 

 The African countries selected for the survey were based on the fact that they are signatories to several international legal instruments advocating against IPV. The Asia country (India) selected was based on the similarities it has with Nigeria. India is reported to be the most populated in the Asian continent and Nigeria is the most populated country in the African continent. They both have diversity in ethnicity, linguistics, culture, and religion, and they are categorised as developing countries that operate common law systems, as well as colonised by the same British colonial master. Therefore, these similarities make it worthy of a comparative survey.

**An analysis of domestic violence incidents in Africa and other selected countries**

 Under this subheading, the authors projected a legal framework around domestic violence as it were in traditional societies and failed to project the current move by different countries to ameliorate the menace of domestic violence.

 Domestic violence is no longer a hush-hush issue as it was in the past. The culture of silence over domestic violence has been broken, many people are beginning to speak out. Similarly, the paper should also look into the roles of some of the treaties many of the aforementioned African counties are signatories to that have also helped to reduce domestic violence.

 Similarly, some states in Nigeria have also adopted more stringent sanctions and panel codes for some types of gender-based violence, the functionality and effectiveness of some of the laws should also be interrogated.

**Response**

 Recently, IPV is gradually becoming an issue of open discussion, but the culture of silence still remains an issue as society views IPV as a family matter. Meanwhile, most of the African countries mentioned within, specifically Nigeria are signatories to several international legal instruments whose roles are to mitigate IPV and all forms of violence against women. For example, the Protocol to the African Charter on Human and Peoples’ Rights on the Rights of Women in Africa, 2005, signed by 42 of 55 African countries prohibits all forms of violence against women. The convention widely ratified by almost all African countries excluding 2, also protects women against violence and discrimination, the same as the International Convention on Civil and Political Rights, 1966. Significantly, the signed and ratified international legal instruments made a number of the Member states create domestic policies that address the issue of IPV in their respective countries. In Nigeria, for instance, The Violence Against Persons (Prohibition) Act 2015 was adopted to address issues of public and private forms of violence including IPV in the capital city of Nigeria, and it provides remedies for victims and penalties for offenders. Since this policy only applies to the Federal Capital Territory, some other States of the country adopt the policy into their State laws. However, the adoption of the policy is remarkable, but the functionality and effectiveness are yet perceived in the country. Further discussion on IPV and Nigerian States law, could be seen in the latter parts of the study.

 Asian continent with a focus on India as the selected country

 Under this subheading and in the previous one, the authors should justify the reasons for the selection of India among all the Asian countries. Similarly, the criteria used for the selection of some of the countries under discussion should also be made clear.

 The lack of clarity in the justification and criteria could also be because the authors did not dedicate a subheading to address the methodology used in the study, it was only mentioned in the abstract. Therefore there is a need to dedicate a section of the manuscript to explain the methods.

**Response**: 

 The justifications for the selection of India among all other Asian countries among others are domestic abuse affects 70% of women in India physically, sexually, emotionally and economically. According to India's National Crime Records Bureau, recorded cases of crime against women rose by 41% (13,892) in 2021 when compared with 2020 (9,782) records, and 31% of the crimes registered were domestic violence. Sadly, 65% of men in India believe women should accept such kind of abuse in order to hold the family together and that women sometimes deserve to be battered. There is no accurate data on the cases of domestic violence in India because most cases go unreported due to fear of the victim or fear of mockery or embarrassment as well as the pressure not to jeopardise the family’s honour. It is interesting to know that section 498A of the Indian Penal Code 1860 was established to protect women from the cruelty of their husband or their relatives. A subheading has been dedicated to address the methodology used in the study to explain the methods.
